# Potential role of SNP rs2071475 in rheumatoid arthritis and inflammatory bowel disease in the East Asian population: a Mendelian randomization study

**DOI:** 10.1007/s10787-023-01363-1

**Published:** 2023-10-19

**Authors:** Bo Wang, Yongqiang Xiong, Ren Li, Shu Zhang

**Affiliations:** 1 Department of Geriatric Digestive Surgery, The Second Affiliated Hospital of Xi’an Jiaotong University, 157 West 5th Road, Xi’an, 710004 Shaanxi China; 2 Experimental Teaching Center for Clinical Skills, The Second Affiliated Hospital of Xi’an Jiaotong University, Xi’an, China

**Keywords:** Rheumatoid arthritis, Crohn’s disease, Ulcerative colitis, Mendelian randomization, Single nucleotide polymorphisms, rs2071475, HLA-DOB

## Abstract

**Background:**

Previous observational studies have identified an association between rheumatoid arthritis (RA) and inflammatory bowel disease (IBD). However, the causal relationship between RA and IBD in the East Asian population remains uncertain.

**Methods:**

The two-sample Mendelian randomization (MR) analysis was conducted to elucidate the potential causal relationship between RA and IBD. Summary-level data from genome-wide association studies (GWAS) in the East Asian population were utilized, including RA (*n* = 19,190) and IBD (*n* = 6543), including Crohn's disease (CD, *n* = 5409) and ulcerative colitis (UC, *n* = 4853). The inverse variance weighted (IVW) method was employed as the primary analysis, supplemented by weighted median, weighted mode, simple median, MR-Egger, and MR-PRESSO analyses. Sensitivity analyses were conducted to assess the robustness of the results. Genetic data for RA (*n* = 22,515) were utilized to validate the findings in the East Asian population.

**Results:**

The IVW method showed no significant association between genetically predicted RA and overall IBD in the East Asian population (OR = 1.028; 95% CI: 0.935–1.129; *P* = 0.567). The subgroup analysis revealed a positive association between RA and CD (OR = 1.268; 95% CI: 1.108–1.451; *P* < 0.001), while a negative association was observed with UC (OR = 0.839; 95% CI: 0.710–0.993; *P* = 0.041). These findings were supported by another set of RA data. Additionally, an SNP rs2071475 was identified to play an important role in CD and UC.

**Conclusion:**

This study revealed a potential increased susceptibility to CD and a decreased susceptibility to UC in the East Asian population with RA. Furthermore, a key SNP rs2071475 was discovered along with its opposite effects in CD and UC. These findings provide new evidence for research on the corresponding molecular mechanisms and offer insights for clinical management of RA-associated IBD.

**Supplementary Information:**

The online version contains supplementary material available at 10.1007/s10787-023-01363-1.

## Introduction

Rheumatoid arthritis (RA) is a chronic inflammatory joint disease of unknown etiology, which can result in cartilage and skeletal damage, as well as disability. Although primarily affecting the joints, RA should be regarded as a syndrome encompassing extra-articular manifestations, such as rheumatoid nodules, pulmonary involvement, gastrointestinal diseases, and systemic complications (JS et al. 2016). Inflammatory bowel disease (IBD) is a chronic nonspecific inflammatory disorder primarily affecting the gastrointestinal tract, encompassing Crohn's disease (CD) and ulcerative colitis (UC). Crohn’s disease is a recurrent transmural inflammatory disorder of the gastrointestinal mucosa, which involves nearly any part of the gastrointestinal tract in a non-continuous manner, with the terminal ileum being commonly affected. It is often accompanied by complications such as strictures, abscesses, or fistulas. In contrast, ulcerative colitis is limited to the colon and its surrounding areas (Baumgart and Sandborn 2007). Histologically, UC exhibits superficial inflammation limited to the mucosa and submucosa, with cryptitis and crypt abscesses. Microscopic features of CD include thickening of the submucosal layer, transmural inflammation, fissuring ulcers, and non-caseating granulomas (Khor et al. 2011).

Epidemiological studies have indicated an increased risk of autoimmune and inflammatory diseases in patients with IBD, both in individuals with CD and UC, with a higher susceptibility to RA (Cohen et al. 2008; Halling et al. 2017; Yang et al. 2018; Park et al. 2019). Simultaneously, an experimental study about RA has also demonstrated that RA is prone to inducing alterations in the gastrointestinal microbiota, particularly in the early stages of the disease (Zaiss et al. 2021). However, the relationship between RA and IBD remains contentious due to the absence of large-scale randomized controlled trials. A study conducted in a European population revealed a positive correlation between the genetic risk of RA and an increased risk of overall IBD, including CD and UC (Meisinger and Freuer 2022). Nevertheless, whether this causal association exists in East Asian populations remains unknown.

In this study, we conducted a two-sample Mendelian randomization (MR) to evaluate the potential causal relationship between RA and IBD, including CD and UC, in East Asian populations. The results showed a potential increased susceptibility to CD and a decreased susceptibility to UC in patients with RA, and SNP rs2071475 may play pivotal role in this causal relationship.

## Methods

### Study design and methods selection

**Fig. 1:**
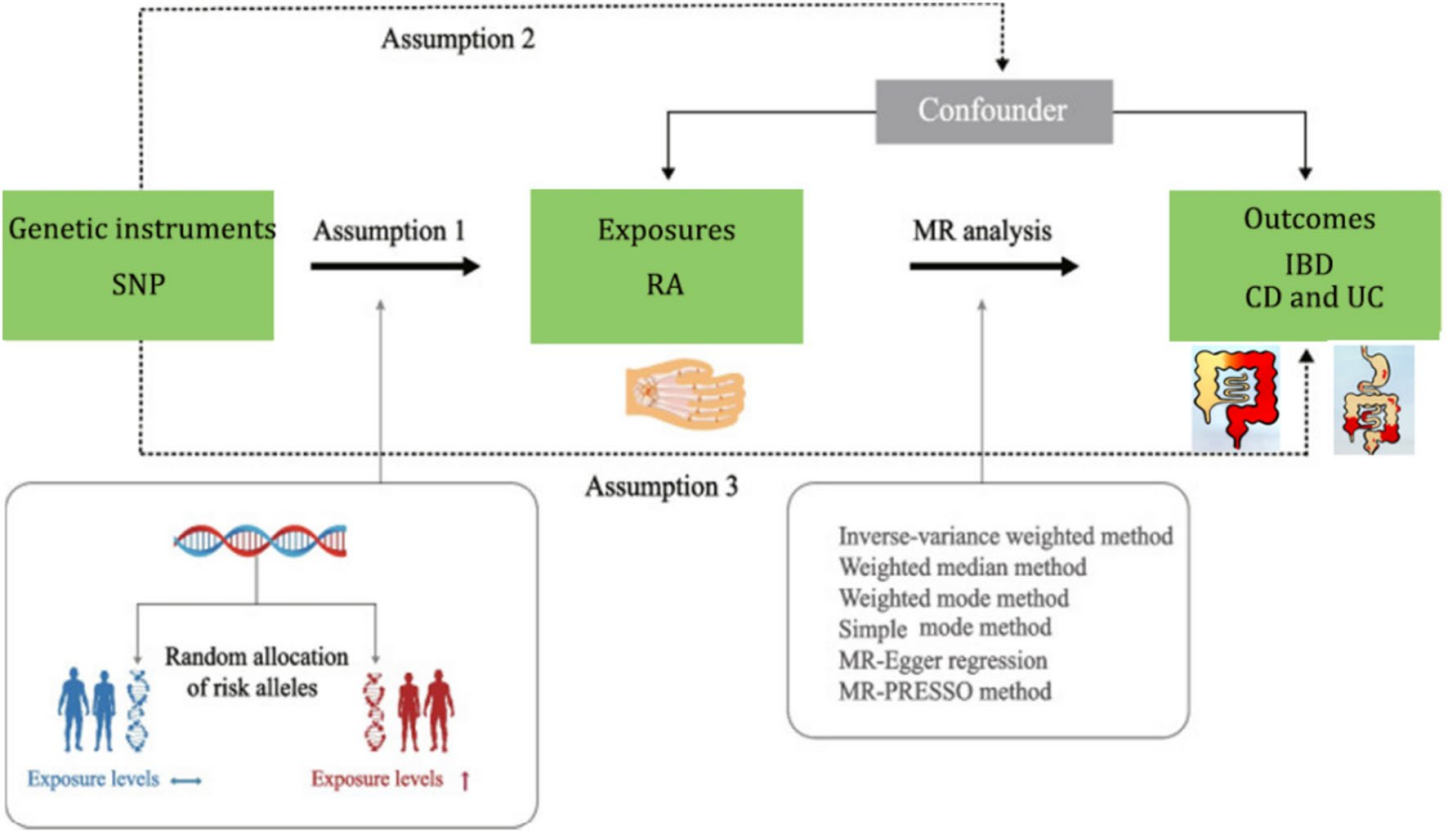
Directed acyclic graph of the MR framework investigating the causal relationship between RA and IBD. *SNPs* single nucleotide polymorphisms; *RA* rheumatoid arthritis; *IBD* inflammatory bowel disease; *CD* Crohn's disease; *UC* ulcerative colitis; *MR* Mendelian randomization

Mendelian randomization (MR) provides a novel approach for causal inference by utilizing genetic variations strongly associated with the exposure factor as instrumental variables (IVs) to infer the causal effects of the exposure on a specific outcome (Davies et al. 2018). Due to the Mendelian inheritance law, which states that alleles are randomly allocated from parents to offspring during gamete formation, genetic variations are not influenced by conventional confounding factors such as environmental influences, socioeconomic factors, or individual behaviors. The temporal relationship between genetic variations and outcomes is therefore plausible. Thus, MR can minimize confounding and reverse causation biases that are commonly encountered in observational studies, offering stronger evidence than observational research (Skrivankova et al. 2021; Davey Smith and Ebrahim 2005). The study design flow chart is shown in Fig. 1.

### Study population selection

This study utilized summary-level data from published studies and databases. Ethical approval and the requirement for informed consent from relevant patients was waived. All genetic data were obtained through a meta-analysis of genome-wide association studies (GWAS) and can be accessible at https://gwas.mrcieu.ac.uk/. The dataset for RA included 19,190 samples (3636 cases and 15,554 controls) from East Asian populations (Okada et al. 2014). The genetic data for IBD were obtained from the International IBD Genetics Consortium (IIBDGC) within GWAS, which included 6543 samples (2824 cases and 3719 controls) from the East Asian population (Liu et al. 2015). In addition, the data of CD with 5409 samples (1690 cases and 3719 controls) and UC with 4853 samples (1134 cases and 3719 controls) were also obtained. Additionally, genetic data from another East Asian population were used to validate the applicability of the results for RA. This validation dataset comprised 22,515 samples (4873 cases and 17,642 controls). Detailed information regarding the GWAS summary data from GWAS for RA and IBD, including CD and UC, can be found in Supplementary Table S1.

### Genetic instrumental variable selection

The single nucleotide polymorphisms (SNPs) serving as instrumental variables (IVs) for RA were obtained from the Japanese Biobank. RA-associated SNPs were selected based on genome-wide significance threshold (*P* < 5 × 10^−8^), indicating substantial correlation with both the SNPs and RA, as well as screening criteria (the linkage disequilibrium *R*^2^ < 0.01 and the length between adjacent SNPs < 1000 kb). Palindromic IVs were excluded after data harmonization, as palindromic SNPs have intermediate allele frequencies. Additionally, an F-statistic threshold exceeding 10 was used to exclude genetic variations as potential IVs (Skrivankova et al. 2021; Burgess and Thompson 2011).

### Statistical analysis

To a valid MR analysis, three assumptions should be met: (1) The genetic variations considered as IVs should be closely associated with the exposure, (2) the genetic variations designated as IVs should not be associated with any confounding factors, and (3) the genetic variations used as IVs should only influence the risk of the outcome through the exposure (Hemani et al. 2018a).

We primarily employed the inverse variance-weighted (IVW) method to assess the impact of exposure (RA) on IBD as well as CD and UC (Bowden et al. 2018). Additionally, we utilized four supplementary analyses to confirm the results, including weighted mode, weighted median, simple mode, and MR-Egger regression (Bowden et al. 2016, 2015; Burgess et al. 2017). To assess whether the MR analysis adheres to the aforementioned three assumptions, we employed Cochran's Q test to assess heterogeneity among SNPs in the IVW and MR-Egger methods (Pereira et al. 2010). MR-Egger regression was used to evaluate the pleiotropy of the IVs (Bowden et al. 2015). MR-PRESSO test was applied to detect and correct for horizontal pleiotropy and leave-one-out testing was performed to examine the influence of specific SNPs (Verbanck et al. 2018). Odds ratios (ORs) with 95% confidence intervals (CIs) were calculated to quantify the relationship between RA and IBD. We drew scatter plots, funnel plots, etc., to clearly visualize the SNP-related RA and IBD risk.

Statistical analysis was conducted using the “TwoSampleMR” (Hemani et al. 2018b) and “MR-PRESSO” (Verbanck et al. 2018) packages in R version 4.2.2, and *p*-value < 0.05 was considered statistically significant.

## Results

### Selection of instrumental variables

We extracted 59 SNPs as IVs from the RA dataset (from the bbj-a-72 project), with a significance level of *P* < 5 × 10^−8^. Additionally, we calculated the F-statistic for each SNP, which ranged from 31.61 to 634.53 and all exceeded 10. This indicates that the IVs are unlikely to be influenced by instrumental bias and are in accordance with the first hypothesis. The details including p-values, beta coefficients, standard errors (SEs) and effect allele for the association between SNPs and RA of the selected IVs are provided in Supplementary Table S2. Finally, for different outcome events, including IBD, CD, and UC, we selected 11/11/10 SNPs, respectively, as genetic instruments for the MR analysis. The information regarding the RA-related genetic variants and their effects on IBD, CD, and UC can be found in Tables 1, 2, 3.

**Table 1:** Characteristic of the RA-related genetic variants and their effects on IBD (11 SNPs)

SNP	Chr	Position	EA	SNPs-RA			SNPs-IBD		
				*β*	SE	*P*-value	*β*	SE	*P*-value
rs10946216	6	167,538,897	C	– 0.24297	0.02598	8.52904E-21	0.0150464	0.0360277	0.676218
rs11889341	2	191,943,742	T	0.15935	0.02743	6.28131E-09	0.00765701	0.0390901	0.844702
rs12174774	6	31,268,965	T	0.26113	0.02677	1.737E-22	–0.0120967	0.0380985	0.750857
rs2071475	6	32,782,387	A	0.35326	0.02741	5.10152E-38	0.0227278	0.0400169	0.570068
rs2240339	1	17,674,108	T	– 0.1833	0.02557	7.68599E-13	0.0119186	0.0361988	0.741964
rs2847266	18	12,773,338	T	– 0.20953	0.03301	2.17485E-10	–0.0549889	0.0351546	0.117815
rs29243	6	29,599,102	A	– 0.43617	0.05054	6.13338E-18	–0.0594563	0.06484	0.359172
rs653520	6	138,145,552	T	– 0.20787	0.03672	1.5144E-08	–0.0522348	0.0557056	0.348404
rs7749924	6	30,797,991	T	0.4588	0.04354	5.74778E-26	–0.0246879	0.0490562	0.614787
rs9380069	6	28,203,300	G	0.2022	0.02931	5.21315E-12	0.0115321	0.0429227	0.788185
rs9494892	6	138,223,489	T	0.29808	0.04647	1.41211E-10	–0.0511752	0.0738841	0.488558

*RA* rheumatoid arthritis; *IBD* inflammatory bowel disease; *SNP* single nucleotide polymorphism; *Chr* chromosome; *EA* effect allele; *SE* standard error

**Table 2:** Characteristic of the RA-related genetic variants and their effects on CD (11 SNPs)

SNP	Chr	Position	EA	SNPs-RA			SNPs-CD		
				*β*	SE	*P*-value	*β*	SE	*P*-value
rs10946216	6	167,538,897	C	– 0.24297	0.02598	8.52904E-21	– 0.00510577	0.0421751	0.903643
rs11889341	2	191,943,742	T	0.15935	0.02743	6.28131E-09	– 0.0119346	0.045915	0.794924
rs12174774	6	31,268,965	T	0.26113	0.02677	1.737E-22	0.161338	0.044128	0.000255941
rs2071475	6	32,782,387	A	0.35326	0.02741	5.10152E-38	0.156929	0.046571	0.000751813
rs2240339	1	17,674,108	T	– 0.1833	0.02557	7.68599E-13	– 0.012537	0.0423958	0.767452
rs2847266	18	12,773,338	T	– 0.20953	0.03301	2.17485E-10	– 0.0748595	0.0414642	0.0710313
rs29243	6	29,599,102	A	– 0.43617	0.05054	6.13338E-18	– 0.105549	0.0791552	0.182383
rs653520	6	138,145,552	T	– 0.20787	0.03672	1.5144E-08	– 0.0679129	0.0650216	0.29629
rs7749924	6	30,797,991	T	0.4588	0.04354	5.74778E-26	0.00279163	0.0580337	0.961634
rs9380069	6	28,203,300	G	0.2022	0.02931	5.21315E-12	0.0603802	0.0502469	0.22949
rs9494892	6	138,223,489	T	0.29808	0.04647	1.41211E-10	0.0272293	0.0844546	0.747137

*RA* rheumatoid arthritis; *CD* Crohn's disease; *SNP* single nucleotide polymorphism; *Chr* chromosome; *EA* effect allele; *SE* standard error

**Table 3:** Characteristic of the RA-related genetic variants and their effects on UC (10 SNPs)

SNP	Chr	Position	EA	SNPs– RA			SNPs– UC		
				*β*	SE	*P*-value	β	SE	*P*-value
rs10946216	6	167,538,897	C	– 0.24297	0.02598	8.52904E-21	0.0531022	0.048559	0.274127
rs11889341	2	191,943,742	T	0.15935	0.02743	6.28131E-09	0.0442206	0.0521982	0.396874
rs2071475	6	32,782,387	A	0.35326	0.02741	5.10152E-38	– 0.191489	0.0563682	0.000675134
rs2240339	1	17,674,108	T	– 0.1833	0.02557	7.68599E-13	0.0418694	0.0487626	0.390529
rs2847266	18	12,773,338	T	– 0.20953	0.03301	2.17485E-10	– 0.0459502	0.0471971	0.330235
rs29243	6	29,599,102	A	– 0.43617	0.05054	6.13338E-18	– 0.00113239	0.0853129	0.98941
rs653520	6	138,145,552	T	– 0.20787	0.03672	1.5144E-08	– 0.0344012	0.0750614	0.646717
rs7749924	6	30,797,991	T	0.4588	0.04354	5.74778E-26	– 0.0563608	0.0664721	0.396477
rs9380069	6	28,203,300	G	0.2022	0.02931	5.21315E-12	– 0.076021	0.0592147	0.199151
rs9494892	6	138,223,489	T	0.29808	0.04647	1.41211E-10	– 0.164131	0.10345	0.112599

*RA* rheumatoid arthritis; *UC* ulcerative colitis; *SNP* single nucleotide polymorphism; *Chr* chromosome; *EA*, effect allele; *SE* standard error

### The effect of RA on IBD, CD and UC

**Fig. 2:**
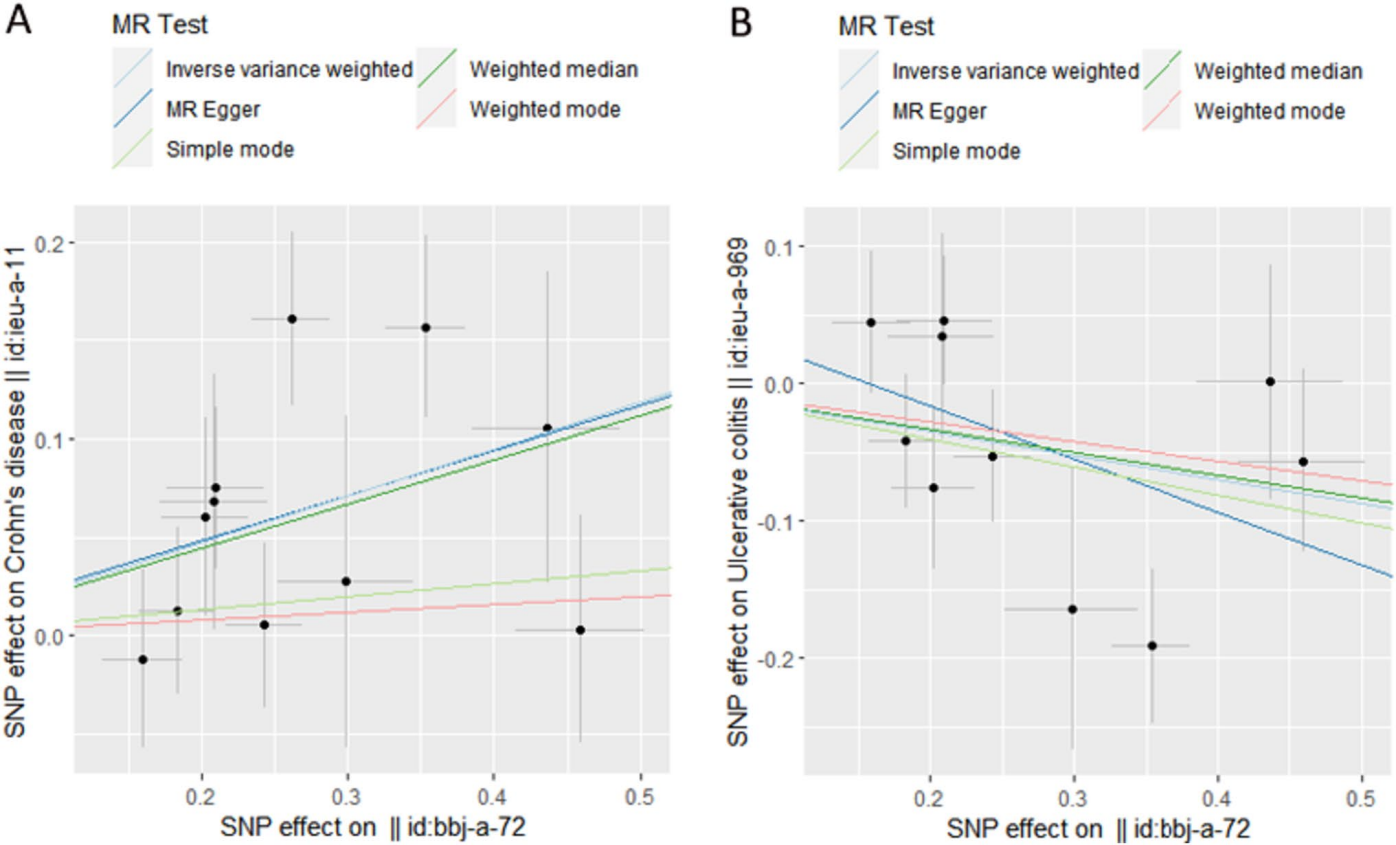
Scatter plots showing the causal effect of SNPs on RA (bbj–a–72) against the effects on CD(A) and UC(B). *SNP* single nucleotide polymorphisms; *MR* Mendelian randomization

According to the IVW analysis, there is no significant causal effect (OR = 1.028; 95% CI: 0.935–1.129; *P* = 0.567) between genetic liability to RA and IBD in the East Asian population (Table 4). However, in the subsequent analysis focusing on the two subtypes, CD and UC, we observed distinct causal associations. Specifically, RA showed a positive correlation with CD (OR = 1.268; 95% CI: 1.108–1.451; *P* < 0.001), while a negative correlation (OR = 0.839; 95% CI: 0.710–0.993; *P* = 0.041) was found between RA and UC (Table 4), and these was visualized in the scatter plot (Fig. 2). This suggests that the presence of RA may serve as a risk factor for CD occurrence in the East Asian population, while acting as a protective factor for UC development. The results obtained from the weighted median method also support these findings.

**Table 4:** Effect estimates of the associations between RA and IBD in East Asian populations

Exposure GWAS ID	Outcome GWAS ID	Method	SNPs(N)	OR	95CI%	MR *P*–value	Heterogeneity Q/P–value	Pleiotropy *P*–value
RA (bbj– a– 72)	IBD (ieu-a-293)	IVW	11	1.028	0.935–1.129	0.567	5.389/0.864	
		MR Egger	11	0.97	0.725–1.298	0.843	5.221/0.815	0.690^a^
		Weighted median	11	1.004	0.888–1.135	0.947		
		Simple mode	11	0.985	0.813–1.194	0.883		
		Weighted mode	11	0.975	0.821–1.159	0.78		
		MR– PRESSO	11	–	–	–		0.871^b^
	CD (ieu-a-11)	IVW	11	1.268	1.108–1.451	< 0.001	14.912/0.135	
		MR Egger	11	1.257	0.807–1.958	0.338	14.909/0.093	0.969^a^
		Weighted median	11	1.251	1.049–1.492	0.013		
		Simple mode	11	1.069	0.761–1.500	0.71		
		Weighted mode	11	1.039	0.728–1.484	0.836		
		MR– PRESSO	11	–	–	–		0.117^b^
	UC (ieu-a-969)	IVW	10	0.839	0.710–0.993	0.041	13.838/0.128	
		MR Egger	10	0.68	0.415–1.114	0.165	12.591/0.127	0.399^a^
		Weighted median	10	0.847	0.695–1.003	0.101		
		Simple mode	10	0.815	0.586–1.135	0.028		
		Weighted mode	10	0.868	0.655–1.150	0.349		
		MR– PRESSO	10	–	–	–		0.141^b^

*RA* rheumatoid arthritis; *IBD* inflammatory bowel disease, *CD* Crohn's disease, *UC* ulcerative colitis; *SNP* single nucleotide polymorphism; *OR* odds ratio; *CI* confidence interval; *IVW* inverse–variance–weighted; *MR*, Mendelian randomization; MR– PRESSO, MR pleiotropy residual sum and outlier

a: *p*–value of the intercept from MR Egger regression analysis

b: *p*–value of MR–PRESSO global test

### Sensitivity analysis

**Fig. 3:**
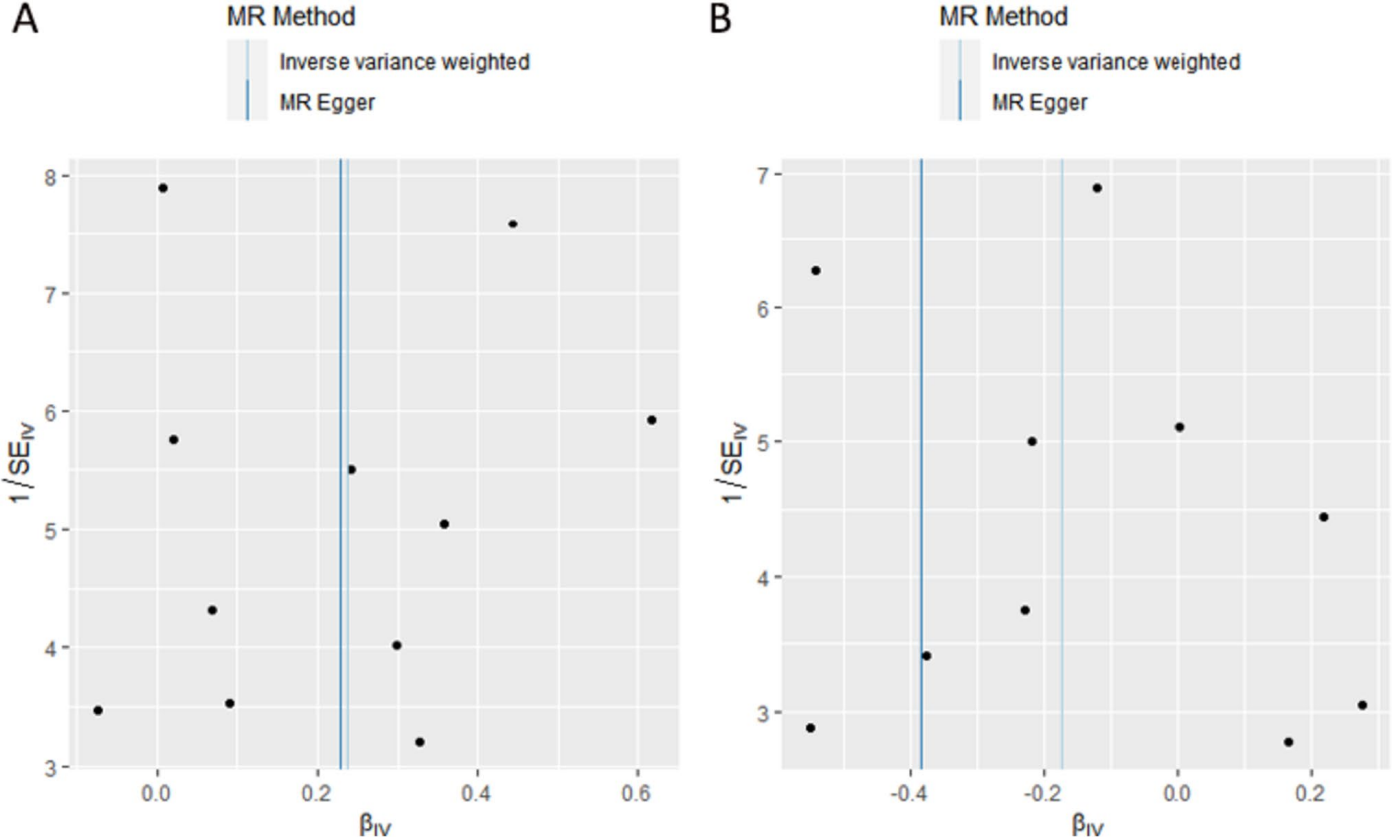
Funnel plots showing no significant heterogeneity among the SNPs of CD(A) and UC(B). *SE* standard error

**Fig. 4:**
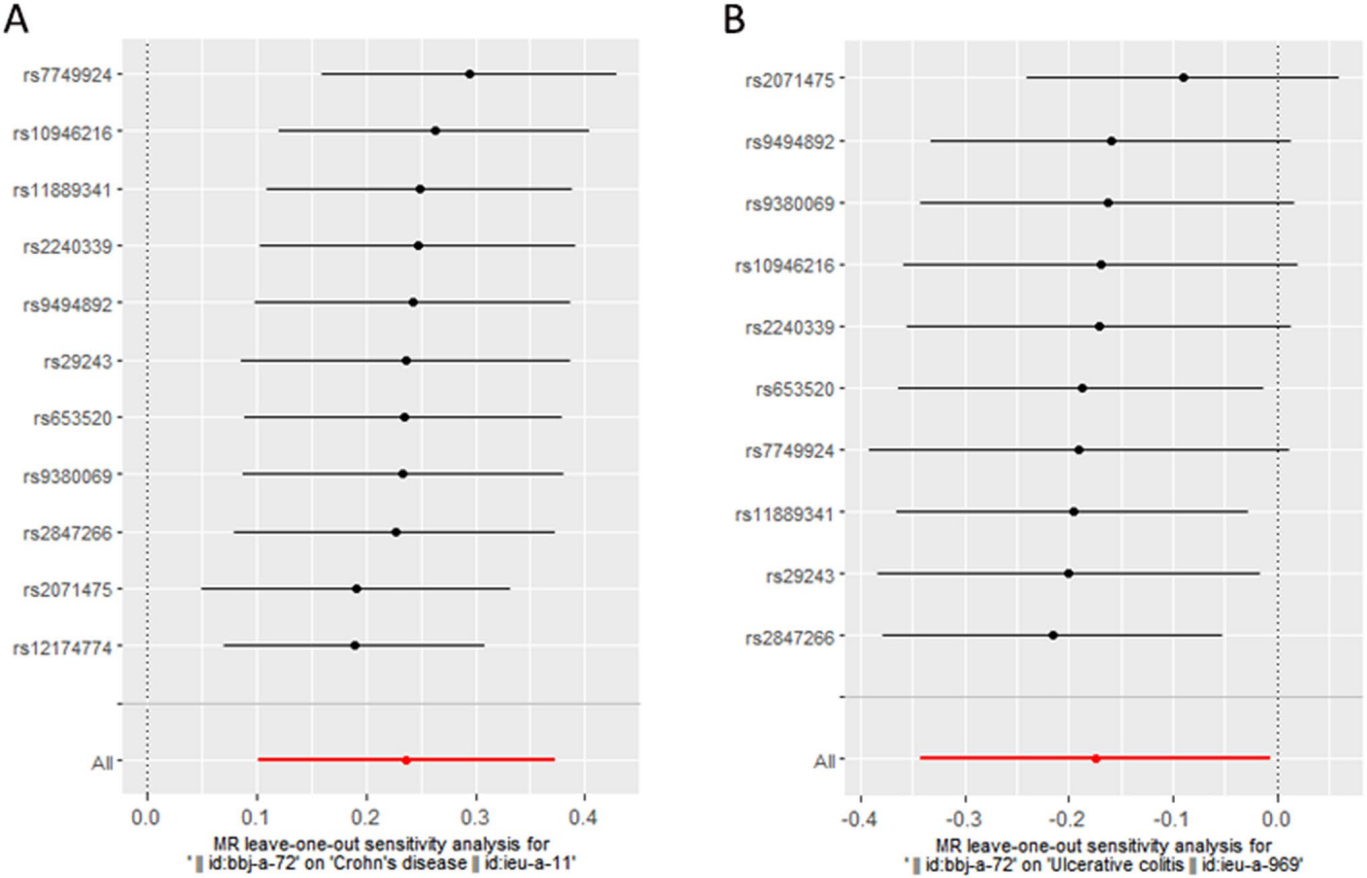
The Forest plot of leave-one-out sensitivity analysis showing the impact of each SNP on the overall causal estimate to CD(A) and UC(B)

For the stability of the results, the Cochran's Q test indicated no significant heterogeneity under the influence of SNPs for both CD and UC (CD: Q = 14.912, *P* = 0.135; UC: *Q* = 13.838, *P* = 0.128), and were shown in the funnel plot (Fig. 3). The intercept *p*-values obtained from the MR-Egger method were 0.969 and 0.399 for CD and UC, respectively, both greater than 0.05, indicating the absence of horizontal pleiotropy in the IVs. The results of MR-PRESSO method also supported this conclusion (Table 4). Additionally, the leave-one-out sensitivity analysis was conducted to assess the impact of each SNP on the overall causal estimate. No significant changes in the estimated causal effects were observed when individual SNPs were excluded (Fig. 4).

To validate our conclusions, we employed genetic data related to RA from another East Asian population (ieu-a-831) for verification. A total of 53 RA-associated SNPs were included as IVs, and the F-statistic was also examined, the detail information can be found in Supplementary Table S3. Due to palindromic with intermediate allele frequencies, one SNP (rs210180) was removed from the relevant MR studies after data harmonization. Information regarding RA-associated genetic variants and their effects on IBD, CD, and UC can be found in Supplementary Table S4–S6. The final IVW analysis yielded similar results, indicating no significant association between RA and overall IBD (OR = 1.102; 95% CI: 0.980–1.239; *P* = 0.104), a positive correlation (OR = 1.202; 95% CI: 1.047–1.381; *P* = 0.009) between RA and CD, and a negative correlation (OR = 0.852; 95% CI: 0.741–0.980; *P* = 0.025) between RA and UC (Table 5). The scatter plot can be found in Fig. S1. Sensitivity analysis also indicated the absence of heterogeneity and horizontal pleiotropy for these SNPs (Figs. S2, S3).

**Table 5:** Verification of the associations between RA and IBD in East Asian populations

Exposure GWAS ID	Outcome GWAS ID	Method	SNPs(N)	OR	95 CI%	MR *P*–value	Heterogeneity Q/P–value	Pleiotropy *P*–value
RA ieu–a–831	IBD (ieu-a-293)	IVW	10	1.102	0.980–1.239	0.104	5.626/0.777	
		MR Egger	10	1.19	0.780–1.753	0.471	5.536/0.699	0.772^a^
		Weighted median	10	1.117	0.953–1.309	0.171		
		Simple mode	10	1.227	0.948–1.588	0.154		
		Weighted mode	10	1.211	0.948–1.546	0.161		
		MR– PRESSO	10	–	–	–		0.793^b^
	CD (ieu-a-11)	IVW	10	1.202	1.047–1.381	0.009	3.670/0.932	
		MR Egger	10	1.397	0.859–2.272	0.215	3.272/0.916	0.546^a^
		Weighted median	10	1.235	1.028–1.484	0.024		
		Simple mode	10	1.335	1.008–1.766	0.074		
		Weighted mode	10	1.325	1.003–1.750	0.079		
		MR– PRESSO	10	–	–	–		0.936^b^
	UC (ieu-a-969)	IVW	12	0.852	0.741–0.980	0.025	18.363/0.073	
		MR Egger	12	0.669	0.519–0.861	0.011	12.577/0.248	0.058^a^
		Weighted median	12	0.804	0.683–0.945	0.008		
		Simple mode	12	0.824	0.587–1.158	0.288		
		Weighted mode	12	0.753	0.623–0.910	0.013		
		MR– PRESSO	12	–	–	–		0.071^b^

*RA* rheumatoid arthritis; *IBD* inflammatory bowel disease, *CD* Crohn's disease, *UC* ulcerative colitis; *SNP* single nucleotide polymorphism; *OR* odds ratio; *CI* confidence interval; *IVW* inverse–variance–weighted; *MR* Mendelian randomization; *MR–PRESSO* MR pleiotropy residual sum and outlier

a: *p*–value of the intercept from MR Egger regression analysis

b: *p*–value of MR– PRESSO global test

## Discussion

As far as we know, this is the first two-sample MR study to comprehensively evaluate the causal relationship between genetic susceptibility to RA on the risk of developing IBD in the East Asian population. By selecting reliable SNPs as instrumental variables (IVs), our findings suggest that genetically predicted RA is significantly associated with an increased incidence of CD and a decreased incidence of UC in the East Asian population.

Previous observational studies have suggested a relationship between RA and IBD, with some prospective studies reporting a higher incidence of RA in patients with IBD (Halling et al. 2017; Yang et al. 2018). Furthermore, one study indicated that RA may potentially induce the occurrence of gut dysbiosis and increase the likelihood of developing IBD (Zaiss et al. 2021). However, these studies have certain limitations. Due to the inherent limitations of observational study designs, current epidemiological research has not adequately assessed the influence of potential confounding variables, such as study duration, variations in different screening programs, environmental exposures, and data collection methods, which could potentially distort the underlying association between RA and IBD. Therefore, drawing a definitive conclusion may be insufficient at this stage.

Mendelian randomization (MR) is a novel approach that utilizes genetic variants as instrumental variables to determine the impact of certain exposures on outcomes (Davies et al. 2018; Skrivankova et al. 2021). Firstly, genetic influences are relatively stable and largely unaffected by environmental factors. Additionally, MR employs stringent quality control criteria and analytical methods, utilizing various models to examine causal effects. Therefore, MR has the potential to overcome limitations associated with traditional observational studies and generate reliable research findings (Davies et al. 2018; Davey Smith and Hemani 2014; Smith and Ebrahim 2003).

Our study found no association between RA and the overall incidence of IBD in the East Asian population. However, interestingly, we observed a higher incidence of CD and a lower incidence of UC among RA patients. It is well known that CD and UC are two subtypes of IBD, both characterized by non-specific inflammation, but they also exhibit some differences in terms of pathology and histology. Further analysis of the genetic variants associated with RA revealed a key SNP, rs2071475. While this SNP showed a strong association with RA (β = 0.35326, *P* = 5.10152E-38), it exhibited completely opposite effects in CD (SNP-CD: *β* = 0.156929, *P* < 0.001) and UC (SNP-UC: *β* = − 0.191489, *P* < 0.001), the data can be found in Tables 2 and 3. The reference SNP report for rs2071475 indicates a close association with HLA-DOB (accessible at https://www.ncbi.nlm.nih.gov/snp/rs2071475). A large-scale meta-analysis has identified that HLA-DOB is significantly upregulated in RA (Afroz et al. 2017). Previous researches have indicated that human leukocyte antigen HLA-DOB, which can influence several alleles involved in antigen presentation, plays a crucial role in viral infections (Denzin et al. 2017; Han et al. 2020). Simultaneously, a study involving transcriptome analysis of the colon found that HLA-DOB was significantly upregulated only in patients with CD, while there was no significant change in patients with UC (Yang et al. 2019). And this study suggests that virus-induced autoimmunity may represent a hypothesis for IBD, particularly CD. This provides a plausible explanation for the differential impact of RA on the incidence of CD and UC observed in our study, possibly due to variations in HLA-DOB expression.

Additionally, some studies have found that Toll-like receptor 4 (TLR4) and its associated innate immune pathways, play a more significant role in UC (Yang et al. 2019; Pastille et al. 2021). Furthermore, another case–control study revealed a significant association between TLR4 polymorphisms and the risk of IBD in individuals of Caucasians (Wang et al. 2019). This also explains why in another study conducted on a European population, patients with rheumatoid arthritis exhibited an increased risk of overall IBD, including both CD and UC (Meisinger and Freuer 2022).

One advantage of our study is that, for the first time, we utilized a two-sample MR approach to assess the impact of RA on IBD, including CD and UC in the East Asian population. Additionally, we identified a significant SNP, rs2071475, that plays a crucial role in CD and UC, offering novel insights into their pathogenesis, diagnosis, and treatment, which hold strong clinical significance. Furthermore, compared to traditional observational study designs, the MR approach is less susceptible to confounding factors, providing more reliable evidence. The consistent and robust causal estimates were further supported by sensitivity analyses. One limitation of our study is the lack of specific clinical data, such as age, gender, duration, activity levels, and treatment modalities, related to RA patients, which hinders further subgroup analyses. Therefore, further research is needed to validate the precise causal relationship between RA and IBD.

## Conclusion

Overall, this MR study reveals a causal association between RA and IBD, specifically an increased risk of CD and a decreased risk of UC among RA patients in the East Asian population. These findings pave the way for further exploration of the underlying molecular mechanisms, clinical implications, strengthened epidemiological surveillance, and informed public health decision-making.

### Supplementary Information

Below is the link to the electronic supplementary material.

Supplementary file1 (DOCX 4509 KB)

## Data Availability

The datasets generated during and/or analyzed during the current study are available at https://gwas.mrcieu.ac.uk/.

## References

[CR1] Afroz S, Giddaluru J, Vishwakarma S, Naz S, Khan AA, Khan N (2017). A comprehensive gene expression meta-analysis identifies novel immune signatures in rheumatoid arthritis patients. Front Immunol.

[CR2] Baumgart DC, Sandborn WJ (2007). Inflammatory bowel disease: clinical aspects and established and evolving therapies. Lancet.

[CR3] Bowden J, Davey Smith G, Burgess S (2015). Mendelian randomization with invalid instruments: effect estimation and bias detection through Egger regression. Int J Epidemiol.

[CR4] Bowden J, Davey Smith G, Haycock PC, Burgess S (2016). Consistent estimation in mendelian randomization with some invalid instruments using a weighted median estimator. Genet Epidemiol.

[CR5] Bowden J, Spiller W, Del Greco MF, Sheehan N, Thompson J, Minelli C, Davey Smith G (2018). Improving the visualization, interpretation and analysis of two-sample summary data mendelian randomization via the radial plot and radial regression. Int J Epidemiol.

[CR6] Burgess S, Thompson SG (2011). Avoiding bias from weak instruments in Mendelian randomization studies. Int J Epidemiol.

[CR7] Burgess S, Bowden J, Fall T, Ingelsson E, Thompson SG (2017). Sensitivity analyses for robust causal inference from mendelian randomization analyses with multiple genetic variants. Epidemiology.

[CR8] Cohen R, Robinson D, Paramore C, Fraeman K, Renahan K, Bala M (2008). Autoimmune disease concomitance among inflammatory bowel disease patients in The United States, 2001–2002. Inflamm Bowel Dis.

[CR9] Davey Smith G, Ebrahim S (2005). What can mendelian randomisation tell us about modifiable behavioural and environmental exposures?. BMJ.

[CR10] Davey Smith G, Hemani G (2014). Mendelian randomization: genetic anchors for causal inference in epidemiological studies. Hum Mol Genet.

[CR11] Davies NM, Holmes MV, Davey Smith G (2018). Reading Mendelian randomisation studies: a guide, glossary. And Checklist for Clinicians Bmj.

[CR12] Denzin LK, Khan AA, Virdis F, Wilks J, Kane M, Beilinson HA, Dikiy S, Case LK, Roopenian D, Witkowski M, Chervonsky AV, Golovkina TV (2017). Neutralizing antibody responses to viral infections are linked to the non-classical MHC class II gene H2-Ob. Immunity.

[CR13] Halling ML, Kjeldsen J, Knudsen T, Nielsen J, Hansen LK (2017). Patients with inflammatory bowel disease have increased risk of autoimmune and inflammatory diseases. World J Gastroenterol.

[CR14] Han J, Chen C, Wang C, Qin N, Huang M, Ma Z, Zhu M, Dai J, Jiang Y, Ma H, Jin G, Shen H, Hu Z (2020). Transcriptome-wide association study for persistent hepatitis B virus infection and related hepatocellular carcinoma. Liver Int.

[CR15] Hemani G, Bowden J, Davey Smith G (2018). Evaluating the potential role of pleiotropy in Mendelian randomization studies. Hum Mol Genet.

[CR16] Hemani G, Zheng J, Elsworth B, Wade KH, Haberland V, Baird D, Laurin C, Burgess S, Bowden J, Langdon R, Tan VY, Yarmolinsky J, Shihab HA, Timpson NJ, Evans DM, Relton C, Martin RM, Davey Smith G, Gaunt TR, Haycock PC (2018). The Mr-base platform supports systematic causal inference across the human phenome. Elife.

[CR17] Js, S., D, A. & Ib, M (2016) Rheumatoid Arthritis. Lancet 388:2023-2038 10.1016/S0140-6736(16)30173-8 27156434

[CR18] Khor B, Gardet A, Xavier RJ (2011). Genetics and pathogenesis of inflammatory bowel disease. Nature.

[CR19] Liu JZ, Van Sommeren S, Huang H, Ng SC, Alberts R, Takahashi A, Ripke S, Lee JC, Jostins L, Shah T, Abedian S, Cheon JH, Cho J, Dayani NE, Franke L, Fuyuno Y, Hart A, Juyal RC, Juyal G, Kim WH, Morris AP, Poustchi H, Newman WG, Midha V, Orchard TR, Vahedi H, Sood A, Sung JY, Malekzadeh R, Westra HJ, Yamazaki K, Yang SK, Barrett JC, Alizadeh BZ, Parkes M, Bk T, Daly MJ, Kubo M, Anderson CA, Weersma RK (2015). Association analyses identify 38 susceptibility loci for inflammatory bowel disease and highlight shared genetic risk across populations. Nat Genet.

[CR20] Meisinger C, Freuer D (2022). Rheumatoid arthritis and inflammatory bowel disease: a bidirectional two-sample Mendelian randomization study. Semin Arthritis Rheum.

[CR21] Okada Y, Wu D, Trynka G, Raj T, Terao C, Ikari K, Kochi Y, Ohmura K, Suzuki A, Yoshida S, Graham RR, Manoharan A, Ortmann W, Bhangale T, Denny JC, Carroll RJ, Eyler AE, Greenberg JD, Kremer JM, Pappas DA, Jiang L, Yin J, Ye L, Su DF, Yang J, Xie G, Keystone E, Westra HJ, Esko T, Metspalu A, Zhou X, Gupta N, Mirel D, Stahl EA, Diogo D, Cui J, Liao K, Guo MH, Myouzen K, Kawaguchi T, Coenen MJ, Van Riel PL, Van De Laar MA, Guchelaar HJ, Huizinga TW, Dieudé P, Mariette X, Bridges SL, Zhernakova A, Toes RE, Tak PP, Miceli-Richard C, Bang SY, Lee HS, Martin J, Gonzalez-Gay MA, Rodriguez-Rodriguez L, Rantapää-Dahlqvist S, Arlestig L, Choi HK, Kamatani Y, Galan P, Lathrop M, Eyre S, Bowes J, Barton A, De Vries N, Moreland LW, Criswell LA, Karlson EW, Taniguchi A, Yamada R, Kubo M, Liu JS, Bae SC, Worthington J, Padyukov L, Klareskog L, Gregersen PK, Raychaudhuri S, Stranger BE, De Jager PL, Franke L, Visscher PM, Brown MA, Yamanaka H, Mimori T, Takahashi A, Xu H, Behrens TW, Siminovitch KA, Momohara S, Matsuda F, Yamamoto K, Plenge RM (2014). Genetics of rheumatoid arthritis contributes to biology and drug discovery. Nature.

[CR22] Park SW, Kim TJ, Lee JY, Kim ER, Hong SN, Chang DK, Yang M, Kim S, Shin MH, Kim YH (2019). Comorbid immune-mediated diseases in inflammatory bowel disease: a nation-wide population-based study. Aliment Pharmacol Ther.

[CR23] Pastille E, Faßnacht T, Adamczyk A, Ngo Thi Phuong N, Buer J, Westendorf AM (2021). Inhibition of Tlr4 signaling impedes tumor growth in colitis-associated colon cancer. Front Immunol.

[CR24] Pereira TV, Patsopoulos NA, Salanti G, Ioannidis JP (2010). Critical interpretation of Cochran's Q test depends on power and prior assumptions about heterogeneity. Res Synth Methods.

[CR25] Skrivankova VW, Richmond RC, Woolf BAR, Davies NM, Swanson SA, Vanderweele TJ, Timpson NJ, Higgins JPT, Dimou N, Langenberg C, Loder EW, Golub RM, Egger M, Davey Smith G, Richards JB (2021). Strengthening the reporting of observational studies in epidemiology using mendelian randomisation (Strobe-Mr): explanation and elaboration. BMJ.

[CR26] Smith GD, Ebrahim S (2003). 'Mendelian randomization': can genetic epidemiology contribute to understanding environmental determinants of disease?. Int J Epidemiol.

[CR27] Verbanck M, Chen CY, Neale B, Do R (2018). Detection of widespread horizontal pleiotropy in causal relationships inferred from mendelian randomization between complex traits and diseases. Nat Genet.

[CR28] Wang H, Zhou S, Zhang J, Lei S, Zhou J (2019). Correlations between tlr polymorphisms and inflammatory bowel disease: a meta-analysis of 49 case-control studies. Immunol Res.

[CR29] Yang BR, Choi NK, Kim MS, Chun J, Joo SH, Kim H, Lee J (2018). Prevalence of extraintestinal manifestations in Korean inflammatory bowel disease patients. PLoS ONE.

[CR30] Yang L, Tang S, Baker SS, Arijs I, Liu W, Alkhouri R, Lan P, Baker RD, Tang Z, Ji G, Rutgeerts P, Vermeire S, Zhu R, Zhu L (2019). Difference in pathomechanism between Crohn's disease and ulcerative colitis revealed by colon transcriptome. Inflamm Bowel Dis.

[CR31] Zaiss MM, Hsin-Jung Joyce W, Mauro D, Schett G, Ciccia F (2021). The gut-joint axis in rheumatoid arthritis. Nat Rev Rheumatol.

