# Supplementary material for: Potential role of SNP rs2071475 in rheumatoid arthritis and inflammatory bowel disease in the East Asian population: a Mendelian randomization study
Source: Inflammopharmacology. 2023 Oct 19;32(1):683–92. doi: 10.1007/s10787-023-01363-1 (PMC10907428; doi:10.1007/s10787-023-01363-1)
Supplement: Supplementary file 1 — Supplementary file1 (DOCX 4509 KB) [file 10787_2023_1363_MOESM1_ESM.docx]

**Supplementary Materials Content**

Table S1 Details of studies of RA and IBD for East Asian population.

Table S2 Seleciting instrumental variables related to RA from bbj-a-72

Table S3 Seleciting instrumental variables related to RA from ieu-a-831

Table S4 Characteristic of the RA-related genetic variants (ieu-a-831) and effects on IBD (10 SNPs)

Table S5 Characteristic of the RA-related genetic variants (ieu-a-831) and effects on CD (10 SNPs)

Table S6 Characteristic of the RA-related genetic variants (ieu-a-831) and effects on UC (12 SNPs)

Fig. S1 Scatter plots showing the causal effect of SNPs on RA (ieu-a-831) against the estimated effects of SNPs on the risk of CD and UC

Fig. S2 Funnel plots showing no significant heterogeneity among the SNPs of CD and UC.

Fig. S3 The Forest plot of leave-one-out sensitivity analysis showing the impact of each SNP on the overall causal estimate to CD and UC.

**Table S1** Details of studies of RA and IBD for East Asian population.

| **Disease** | **GWAS ID** | **Year** | **First author** | **PMID** | **Sample size** | **n case** | **n control** | **Consortium** |
| --- | --- | --- | --- | --- | --- | --- | --- | --- |
| RA | bbj-a-72 | 2019 | Ishigaki K | 24390342 | 19,190 | 3,636 | 15,554 | NA |
| RA | ieu-a-831 | 2014 | Okada Y | 24390342 | 22,515 | 4,873 | 17,642 | NA |
| IBD | ieu-a-293 | 2015 | Liu | 26192919 | 6,543 | 2,824 | 3,719 | IIBDGC |
| CD | ieu-a-11 | 2015 | Liu | 26192919 | 5,409 | 1,690 | 3,719 | IIBDGC |
| UC | ieu-a-969 | 2015 | Liu | 26192919 | 4,853 | 1,134 | 3,719 | IIBDGC |

GWAS, genome-wide association studies; RA, rheumatoid arthritis; IBD, inflammatory bowel disease; CD, Crohn's disease; UC, ulcerative colitis; IIBDGC, International IBD Genetics Consortium.

**Table S2** Seleciting instrumental variables related to RA from bbj-a-72 (59SNPs)

| **SNP** | **Chr** | **Position** | **EA** | **OA** | **β** | **SE** | **P-value** | **F** |
| --- | --- | --- | --- | --- | --- | --- | --- | --- |
| rs2240339 | 1 | 17674108 | T | C | -0.1833 | 0.02557 | 7.68599E-13 | 51.38820807 |
| rs11889341 | 2 | 191943742 | T | C | 0.15935 | 0.02743 | 6.28131E-09 | 33.74834813 |
| rs9277651 | 6 | 33083840 | G | A | 0.31135 | 0.04744 | 5.27594E-11 | 43.07332316 |
| rs3130165 | 6 | 33130226 | G | C | -0.35421 | 0.03679 | 6.08836E-22 | 92.69622759 |
| rs9262555 | 6 | 31005464 | T | C | -0.46491 | 0.05648 | 1.84544E-16 | 67.75609986 |
| rs17208188 | 6 | 32195005 | T | C | 0.39344 | 0.02905 | 8.43335E-42 | 183.4276277 |
| rs9494892 | 6 | 138223489 | T | G | 0.29808 | 0.04647 | 1.41211E-10 | 41.14530918 |
| rs62401081 | 6 | 24969042 | A | T | -0.4164 | 0.07326 | 1.31807E-08 | 32.30632911 |
| rs7751645 | 6 | 26443614 | G | T | -0.36291 | 0.05454 | 2.85759E-11 | 44.27592096 |
| rs1150697 | 6 | 28175636 | G | C | -0.20675 | 0.02816 | 2.09942E-13 | 53.90458856 |
| rs9380069 | 6 | 28203300 | G | A | 0.2022 | 0.02931 | 5.21315E-12 | 47.59163944 |
| rs17596180 | 6 | 33876600 | G | A | 0.28098 | 0.04102 | 7.37225E-12 | 46.92016215 |
| rs56144236 | 6 | 32720219 | A | G | -0.28671 | 0.0412 | 3.4261E-12 | 48.42741075 |
| rs35741362 | 6 | 27007687 | C | T | -0.39525 | 0.059 | 2.09701E-11 | 44.87864479 |
| rs113721192 | 6 | 32682590 | A | G | -0.30946 | 0.04928 | 3.39907E-10 | 39.43371053 |
| rs3129834 | 6 | 30267372 | A | C | 0.41898 | 0.05325 | 3.56862E-15 | 61.90808363 |
| rs9394220 | 6 | 34416388 | G | T | -0.18833 | 0.02998 | 3.34103E-10 | 39.46169684 |
| rs187095086 | 6 | 32368942 | A | G | 0.74898 | 0.03493 | 5.0816E-102 | 459.7727953 |
| rs58667488 | 6 | 32431785 | C | A | 0.69902 | 0.02775 | 4.5709E-140 | 634.5315613 |
| rs2454138 | 6 | 32570401 | A | G | 0.55455 | 0.02817 | 3.17249E-86 | 387.5321389 |
| rs9268182 | 6 | 32276027 | G | T | -0.4995 | 0.0358 | 3.11961E-44 | 194.672646 |
| rs78515650 | 6 | 33800340 | C | T | -0.29523 | 0.03139 | 5.21555E-21 | 88.45824966 |
| rs569785491 | 6 | 31979479 | T | A | 0.67693 | 0.03819 | 2.70271E-70 | 314.1869962 |
| rs2596465 | 6 | 31412948 | C | T | 0.24036 | 0.02866 | 5.07925E-17 | 70.33508531 |
| rs9275818 | 6 | 32690551 | A | G | -0.28671 | 0.04219 | 1.07523E-11 | 46.18135068 |
| **SNP** | **Chr** | **Position** | **EA** | **OA** | **β** | **SE** | **P-value** | **F** |
| rs7760659 | 6 | 25434842 | T | C | -0.3841 | 0.05867 | 5.8776E-11 | 42.86042152 |
| rs29243 | 6 | 29599102 | A | G | -0.43617 | 0.05054 | 6.13338E-18 | 74.48024685 |
| rs72492266 | 6 | 29777453 | T | C | -0.4421 | 0.05031 | 1.52792E-18 | 77.22046192 |
| rs116023803 | 6 | 32378701 | G | A | -0.44577 | 0.0416 | 8.51726E-27 | 114.8246191 |
| rs9461744 | 6 | 32388317 | G | A | -0.3169 | 0.04616 | 6.63285E-12 | 47.13167541 |
| rs2071475 | 6 | 32782387 | A | G | 0.35326 | 0.02741 | 5.10152E-38 | 166.1004688 |
| rs851027 | 6 | 35990875 | G | A | -0.15415 | 0.02675 | 8.2861E-09 | 33.20775264 |
| rs76153210 | 6 | 44284508 | T | C | 0.18734 | 0.02973 | 2.97276E-10 | 39.70737829 |
| rs7749924 | 6 | 30797991 | T | C | 0.4588 | 0.04354 | 5.74778E-26 | 111.0375752 |
| rs858983 | 6 | 27173516 | A | G | 0.38873 | 0.05724 | 1.10688E-11 | 46.12080368 |
| rs9262657 | 6 | 31034426 | A | G | -0.41932 | 0.0455 | 3.10957E-20 | 84.93141524 |
| rs2244020 | 6 | 31347451 | G | A | -0.31315 | 0.02528 | 2.98332E-35 | 153.4442762 |
| rs139395255 | 6 | 31449736 | G | A | 0.49735 | 0.03864 | 6.62217E-38 | 165.672341 |
| rs11965463 | 6 | 32979677 | A | G | -0.47423 | 0.06703 | 1.49211E-12 | 50.05409454 |
| rs3806109 | 6 | 33770370 | G | T | 0.21465 | 0.02843 | 4.3381E-14 | 57.00435897 |
| rs113432940 | 6 | 26256324 | A | T | -0.30834 | 0.04808 | 1.42171E-10 | 41.12735862 |
| rs2859375 | 6 | 28431251 | T | C | -0.40367 | 0.05128 | 3.48819E-15 | 61.96649277 |
| rs12174774 | 6 | 31268965 | T | C | 0.26113 | 0.02677 | 1.737E-22 | 95.15175329 |
| rs10946216 | 6 | 167538897 | C | T | -0.24297 | 0.02598 | 8.52904E-21 | 87.46353253 |
| rs6905149 | 6 | 31944790 | T | C | 0.40246 | 0.03411 | 3.92826E-32 | 139.2137098 |
| rs9258004 | 6 | 29655922 | A | G | -0.32044 | 0.03435 | 1.05925E-20 | 87.02418472 |
| rs1853591 | 6 | 25915405 | G | A | 0.18748 | 0.02825 | 3.16811E-11 | 44.04260368 |
| rs11970475 | 6 | 29526377 | A | G | -0.34106 | 0.0337 | 4.44939E-24 | 102.4240097 |
| rs17212937 | 6 | 32681339 | T | C | 0.72672 | 0.02891 | 2.0749E-139 | 631.8849938 |
| rs145044106 | 6 | 33734241 | C | G | -0.66228 | 0.10612 | 4.3448E-10 | 38.94832611 |
| rs2495964 | 6 | 33919050 | A | G | -0.16136 | 0.0287 | 1.90209E-08 | 31.61025337 |
| rs190669824 | 6 | 44242789 | G | A | 0.21535 | 0.02974 | 4.46581E-13 | 52.4333763 |
| rs653520 | 6 | 138145552 | T | G | -0.20787 | 0.03672 | 1.5144E-08 | 32.046329 |
| rs73432769 | 6 | 30716170 | T | C | -0.46179 | 0.0556 | 1.00138E-16 | 68.98258504 |
| rs1995675 | 6 | 25413828 | A | G | -0.37132 | 0.06145 | 1.51091E-09 | 36.51345333 |
| rs4481423 | 6 | 25720761 | C | T | 0.40764 | 0.0593 | 6.20869E-12 | 47.25461173 |
| rs111508444 | 6 | 29603512 | G | A | -0.45944 | 0.05801 | 2.36374E-15 | 62.72661845 |
| rs10821944 | 10 | 63785089 | T | G | -0.16094 | 0.02609 | 6.9215E-10 | 38.0522057 |
| rs2847266 | 18 | 12773338 | T | C | -0.20953 | 0.03301 | 2.17485E-10 | 40.29038128 |

SNP, single nucleotide polymorphism; Chr, chromosome; EA, effect allele; OA, other allele, SE, standard error.

F were calculated using the following formula: R2(N-2)/(1- R2), where R2 is the proportion of variance in rheumatoid arthritis explained by each instrument. R2 were calculated using the following formula: 2EAF*(1-EAF)*Beta2, where EAF is the minor allele frequency , Beta is the estimated effect on rheumatoid arthritis. And N is the sample size of the GWAS for the rheumatoid arthritis association.

**Table S3** Seleciting instrumental variables related to RA from ieu-a-831 (53SNPs)

| **SNP** | **Chr** | **Position** | **EA** | **OA** | **β** | **SE** | **P-value** | **F** |
| --- | --- | --- | --- | --- | --- | --- | --- | --- |
| rs2240339 | 1 | 17674108 | T | C | -0.18633 | 0.0275227 | 7.70016E-13 | 45.83354988 |
| rs11889341 | 2 | 191943742 | T | C | 0.157004 | 0.0282529 | 6.29999E-09 | 30.88128544 |
| rs653520 | 6 | 138145552 | T | G | -0.210721 | 0.0344834 | 0.000000015 | 37.34181968 |
| rs3129838 | 6 | 30306553 | A | G | 0.41871 | 0.0535505 | 2.99985E-15 | 61.13636399 |
| rs2442731 | 6 | 31318385 | A | G | -0.162519 | 0.0240274 | 5.79963E-11 | 45.75038214 |
| rs2244020 | 6 | 31347451 | G | A | -0.314811 | 0.0260915 | 2.99985E-35 | 145.579996 |
| rs9394220 | 6 | 34416388 | G | T | -0.19062 | 0.0295489 | 3.29997E-10 | 41.61542113 |
| rs2859375 | 6 | 28431251 | T | C | -0.400478 | 0.0535001 | 3.50026E-15 | 56.03355015 |
| rs7749924 | 6 | 30797991 | T | C | 0.457425 | 0.0435614 | 5.70033E-26 | 110.2646075 |
| rs11961777 | 6 | 32379535 | C | T | -0.444686 | 0.0408381 | 8.49963E-27 | 118.5702832 |
| rs11965463 | 6 | 32979677 | A | G | -0.478036 | 0.0651395 | 1.50003E-12 | 53.8557857 |
| rs2495964 | 6 | 33919050 | A | G | -0.162519 | 0.0300467 | 1.89998E-08 | 29.25598476 |
| rs3130165 | 6 | 33130226 | G | C | -0.356675 | 0.0365053 | 6.09958E-22 | 95.46265224 |
| rs7760659 | 6 | 25434842 | T | C | -0.385662 | 0.0560866 | 5.90065E-11 | 47.28195997 |
| rs769174 | 6 | 31551066 | G | C | -0.510826 | 0.0597961 | 5.70033E-19 | 72.97939667 |
| rs10946216 | 6 | 167538897 | C | T | -0.24686 | 0.026033 | 8.49963E-21 | 89.91931971 |
| rs9494892 | 6 | 138223489 | T | G | 0.300105 | 0.047201 | 1.40001E-10 | 40.42444472 |
| rs1853591 | 6 | 25915405 | G | A | 0.18633 | 0.0307724 | 3.19963E-11 | 36.66425205 |
| rs497058 | 6 | 32956720 | C | T | 0.139262 | 0.0265664 | 1.09999E-08 | 27.47893148 |
| rs851027 | 6 | 35990875 | G | A | -0.157004 | 0.0261873 | 8.30004E-09 | 35.94512409 |
| rs35741362 | 6 | 27007687 | C | T | -0.392042 | 0.0599979 | 2.09991E-11 | 42.69658031 |
| rs1150697 | 6 | 28175636 | G | C | -0.210721 | 0.0281995 | 2.09991E-13 | 55.83838146 |
| rs2454138 | 6 | 32570401 | A | G | 0.553885 | 0.0278036 | 3.19963E-86 | 396.8598321 |
| rs6904716 | 6 | 33741289 | G | A | -0.285179 | 0.0304962 | 1E-20 | 87.44674113 |
| rs62401081 | 6 | 24969042 | A | T | -0.415515 | 0.0733883 | 1.29999E-08 | 32.05676972 |
| rs1995675 | 6 | 25413828 | A | G | -0.371064 | 0.062713 | 1.5E-09 | 35.00922449 |
| rs9380069 | 6 | 28203300 | G | A | 0.198451 | 0.0311486 | 5.19996E-12 | 40.59097843 |
| rs11970475 | 6 | 29526377 | A | G | -0.34249 | 0.0321532 | 4.40048E-24 | 113.4612038 |
| rs58667488 | 6 | 32431785 | C | A | 0.693147 | 0.0257898 | 4.6026E-140 | 722.36176 |
| rs9501064 | 6 | 31140445 | T | C | -0.18633 | 0.0307724 | 2.30001E-10 | 36.66425205 |
| rs210180 | 6 | 33485259 | A | T | -0.274437 | 0.0403633 | 2.90001E-11 | 46.22873342 |
| rs72492266 | 6 | 29777453 | T | C | -0.446287 | 0.051591 | 1.50003E-18 | 74.83083318 |
| rs10947453 | 6 | 33806268 | A | G | -0.235722 | 0.0323346 | 1.99986E-14 | 53.14534722 |
| rs2006006 | 6 | 26260875 | G | A | -0.300105 | 0.0510013 | 0.000000002 | 34.62453662 |
| rs9467756 | 6 | 26435359 | A | G | -0.356675 | 0.0552732 | 2.99985E-11 | 41.64051795 |
| rs9258004 | 6 | 29655922 | A | G | -0.314711 | 0.0350003 | 1.10002E-20 | 80.8500536 |
| rs4481423 | 6 | 25720761 | C | T | 0.400478 | 0.0612119 | 6.20012E-12 | 42.80412332 |
| rs9268182 | 6 | 32276027 | G | T | -0.500775 | 0.0355095 | 3.10027E-44 | 198.8823143 |
| rs9271375 | 6 | 32587067 | A | G | 0.451076 | 0.0292795 | 3.80014E-54 | 237.3406509 |
| rs858983 | 6 | 27173516 | A | G | 0.392042 | 0.0569244 | 1.10002E-11 | 47.43165394 |
| rs67068100 | 6 | 31429913 | C | G | 0.527633 | 0.0386607 | 1.59993E-43 | 186.2620908 |
| **SNP** | **Chr** | **Position** | **EA** | **OA** | **β** | **SE** | **P-value** | **F** |
| rs17208188 | 6 | 32195005 | T | C | 0.392042 | 0.0292356 | 8.4004E-42 | 179.8213152 |
| rs9501632 | 6 | 32420599 | T | C | -0.34249 | 0.0359894 | 6.4998E-24 | 90.56211955 |
| rs12234159 | 6 | 31424086 | C | T | 0.356675 | 0.0330811 | 1.59993E-28 | 116.2479909 |
| rs7764819 | 6 | 32680576 | G | T | 0.733969 | 0.0263225 | 2.0989E-139 | 777.5013756 |
| rs29243 | 6 | 29599102 | A | G | -0.430783 | 0.0472302 | 6.09958E-18 | 83.19122962 |
| rs259942 | 6 | 30015167 | T | C | -0.223144 | 0.0349131 | 2.39994E-11 | 40.85014505 |
| rs3734708 | 6 | 44243117 | A | G | 0.215111 | 0.0288325 | 4.49987E-13 | 55.66223455 |
| rs73432769 | 6 | 30716170 | T | C | -0.462035 | 0.0524092 | 1E-16 | 77.72033965 |
| rs9295939 | 6 | 30953968 | G | A | -0.470004 | 0.0572809 | 3.40017E-17 | 67.32609756 |
| rs56313034 | 6 | 33124624 | G | T | -0.307485 | 0.0468533 | 2.60016E-11 | 43.06926887 |
| rs10821944 | 10 | 63785089 | T | G | -0.162519 | 0.0268777 | 6.90001E-10 | 36.56150617 |
| rs2847266 | 18 | 12773338 | T | C | -0.210721 | 0.0344834 | 2.19999E-10 | 37.34181968 |

SNP, single nucleotide polymorphism; Chr, chromosome; EA, effect allele; OA, other allele, SE, standard error.

F were calculated using the following formula: R2(N-2)/(1- R2), where R2 is the proportion of variance in rheumatoid arthritis explained by each instrument. R2 were calculated using the following formula: 2EAF*(1-EAF)*Beta2, where EAF is the minor allele frequency , Beta is the estimated effect on rheumatoid arthritis. And N is the sample size of the GWAS for the rheumatoid arthritis association.

**Table S4**  Characteristic of the RA-related genetic variants (ieu-a-831) and effects on IBD (10 SNPs)

| **SNP** | **Chr** | **EA** | **SNPs-RA** | | | **SNPs-IBD** | | |
| --- | --- | --- | --- | --- | --- | --- | --- | --- |
|  |  |  | **β** | **SE** | **P-value** | **β** | **SE** | **P-value** |
| rs10946216 | 6 | C | -0.24686 | 0.026033 | 8.49963E-21 | 0.0150464 | 0.0360277 | 0.676218 |
| rs11889341 | 2 | T | 0.157004 | 0.0282529 | 6.29999E-09 | 0.00765701 | 0.0390901 | 0.844702 |
| rs2240339 | 1 | T | -0.18633 | 0.0275227 | 7.70016E-13 | 0.0119186 | 0.0361988 | 0.741964 |
| rs259942 | 6 | T | -0.223144 | 0.0349131 | 2.39994E-11 | -0.0620634 | 0.0444018 | 0.162208 |
| rs2847266 | 18 | T | -0.210721 | 0.0344834 | 2.19999E-10 | -0.0549889 | 0.0351546 | 0.117815 |
| rs29243 | 6 | A | -0.430783 | 0.0472302 | 6.09958E-18 | -0.0594563 | 0.06484 | 0.359172 |
| rs3129838 | 6 | A | 0.41871 | 0.0535505 | 2.99985E-15 | 0.0816457 | 0.0722384 | 0.258408 |
| rs653520 | 6 | T | -0.210721 | 0.0344834 | 0.000000015 | -0.0522348 | 0.0557056 | 0.348404 |
| rs9380069 | 6 | G | 0.198451 | 0.0311486 | 5.19996E-12 | 0.0115321 | 0.0429227 | 0.788185 |
| rs9494892 | 6 | T | 0.300105 | 0.047201 | 1.40001E-10 | -0.0511752 | 0.0738841 | 0.488558 |

SNP, single nucleotide polymorphism; Chr, chromosome; EA, effect allele; SE, standard error.

**Table S5** Characteristic of the RA-related genetic variants (ieu-a-831) and effects on CD (10 SNPs)

| **SNP** | **Chr** | **Position** | **EA** | **SNPs-RA** | | | **SNPs-CD** | | |
| --- | --- | --- | --- | --- | --- | --- | --- | --- | --- |
|  |  |  |  | **β** | **SE** | **P-value** | **β** | **SE** | **P-value** |
| rs10946216 | 6 | 167538897 | C | -0.24686 | 0.026033 | 8.49963E-21 | -0.00510577 | 0.0421751 | 0.903643 |
| rs11889341 | 2 | 191943742 | T | 0.157004 | 0.0282529 | 6.29999E-09 | -0.0119346 | 0.045915 | 0.794924 |
| rs2240339 | 1 | 17674108 | T | -0.18633 | 0.0275227 | 7.70016E-13 | -0.012537 | 0.0423958 | 0.767452 |
| rs259942 | 6 | 30015167 | T | -0.223144 | 0.0349131 | 2.39994E-11 | -0.0399454 | 0.0528833 | 0.450038 |
| rs2847266 | 18 | 12773338 | T | -0.210721 | 0.0344834 | 2.19999E-10 | -0.0748595 | 0.0414642 | 0.0710313 |
| rs29243 | 6 | 29599102 | A | -0.430783 | 0.0472302 | 6.09958E-18 | -0.105549 | 0.0791552 | 0.182383 |
| rs3129838 | 6 | 30306553 | A | 0.41871 | 0.0535505 | 2.99985E-15 | 0.124543 | 0.0870381 | 0.152468 |
| rs653520 | 6 | 138145552 | T | -0.210721 | 0.0344834 | 0.000000015 | -0.0679129 | 0.0650216 | 0.29629 |
| rs9380069 | 6 | 28203300 | G | 0.198451 | 0.0311486 | 5.19996E-12 | 0.0603802 | 0.0502469 | 0.22949 |
| rs9494892 | 6 | 138223489 | T | 0.300105 | 0.047201 | 1.40001E-10 | 0.0272293 | 0.0844546 | 0.747137 |

SNP, single nucleotide polymorphism; Chr, chromosome; EA, effect allele; SE, standard error.

**Table S6** Characteristic of the RA-related genetic variants (ieu-a-831) and effects on UC (12 SNPs)

| **SNP** | **Chr** | **Position** | **EA** | **SNPs-RA** | | | **SNPs-UC** | | |
| --- | --- | --- | --- | --- | --- | --- | --- | --- | --- |
|  |  |  |  | **β** | **SE** | **P-value** | **β** | **SE** | **P-value** |
| rs10946216 | 6 | 167538897 | C | -0.24686 | 0.026033 | 8.49963E-21 | 0.0531022 | 0.048559 | 0.274127 |
| rs11889341 | 2 | 191943742 | T | 0.157004 | 0.0282529 | 6.29999E-09 | 0.0442206 | 0.0521982 | 0.396874 |
| rs2240339 | 1 | 17674108 | T | -0.18633 | 0.0275227 | 7.70016E-13 | 0.0418694 | 0.0487626 | 0.390529 |
| rs259942 | 6 | 30015167 | T | -0.223144 | 0.0349131 | 2.39994E-11 | -0.0905181 | 0.0601219 | 0.132124 |
| rs2847266 | 18 | 12773338 | T | -0.210721 | 0.0344834 | 2.19999E-10 | -0.0459502 | 0.0471971 | 0.330235 |
| rs29243 | 6 | 29599102 | A | -0.430783 | 0.0472302 | 6.09958E-18 | -0.00113239 | 0.0853129 | 0.98941 |
| rs3129838 | 6 | 30306553 | A | 0.41871 | 0.0535505 | 2.99985E-15 | 0.0326047 | 0.0950297 | 0.731521 |
| rs653520 | 6 | 138145552 | T | -0.210721 | 0.0344834 | 0.000000015 | -0.0344012 | 0.0750614 | 0.646717 |
| rs7749924 | 6 | 30797991 | T | 0.457425 | 0.0435614 | 5.70033E-26 | -0.0563608 | 0.0664721 | 0.396477 |
| rs7764819 | 6 | 32680576 | G | 0.733969 | 0.0263225 | 2.0989E-139 | -0.260313 | 0.0657409 | 7.36004E-05 |
| rs9380069 | 6 | 28203300 | G | 0.198451 | 0.0311486 | 5.19996E-12 | -0.076021 | 0.0592147 | 0.199151 |
| rs9494892 | 6 | 138223489 | T | 0.300105 | 0.047201 | 1.40001E-10 | -0.164131 | 0.10345 | 0.112599 |

SNP, single nucleotide polymorphism; Chr, chromosome; EA, effect allele; SE, standard error


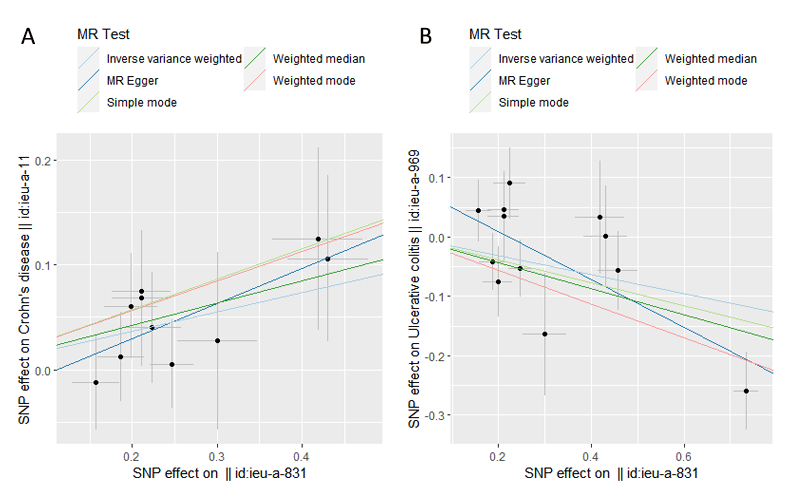


**Fig. S1 Scatter plots showing the causal effect of SNPs on RA (ieu-a-831) against the estimated effects of SNPs on the risk of CD(A) and UC(B)**

SNP, single nucleotide polymorphisms; MR, Mendelian randomization


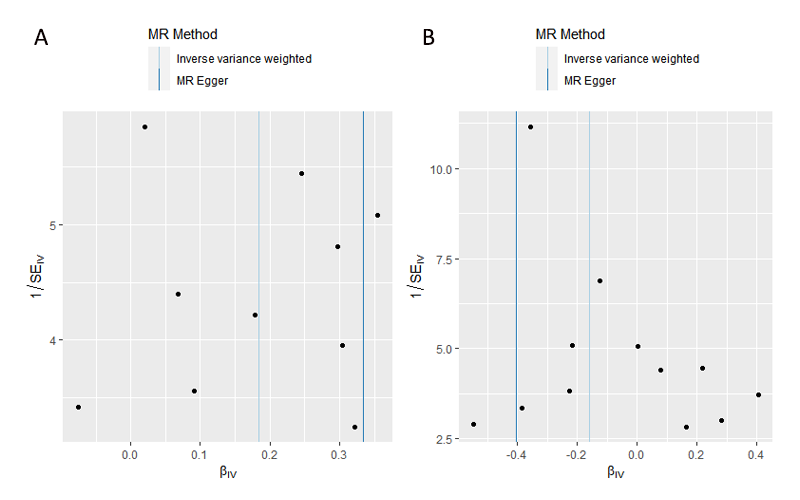


**Fig. S2 Funnel plots showing no significant heterogeneity among the SNPs of CD(A) and UC(B).**

SE, standard error


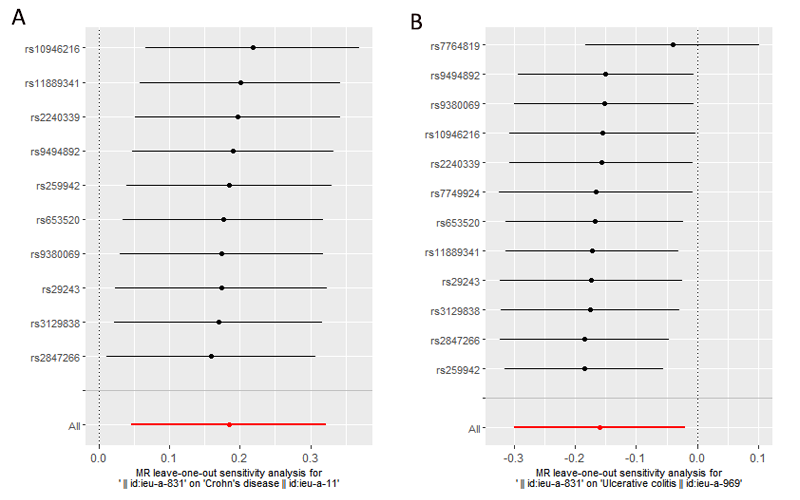


**Fig. S3 The Forest plot of leave-one-out sensitivity analysis showing the impact of each SNP on the overall causal estimate to CD(A) and UC(B).**
